# Matrix Analysis of the Digital Divide in eHealth Services Using Awareness, Want, and Adoption Gap

**DOI:** 10.2196/jmir.1670

**Published:** 2012-02-13

**Authors:** Te-Hsin Liang

**Affiliations:** ^1^Department of Statistics and Information SciencesCollege of ManagementFu Jen Catholic UniversityNew Taipei CountyTaiwan

**Keywords:** Consumer behavior process, digital divide, eHealth, innovation adoption process

## Abstract

**Background:**

The digital divide usually refers to access or usage, but some studies have identified two other divides: awareness and demand (want). Given that the hierarchical stages of the innovation adoption process of a customer are interrelated, it is necessary and meaningful to analyze the digital divide in eHealth services through three main stages, namely, awareness, want, and adoption.

**Objective:**

By following the three main integrated stages of the innovation diffusion theory, from the customer segment viewpoint, this study aimed to propose a new matrix analysis of the digital divide using the awareness, want, and adoption gap ratio (AWAG). I compared the digital divide among different groups. Furthermore, I conducted an empirical study on eHealth services to present the practicability of the proposed methodology.

**Methods:**

Through a review and discussion of the literature, I proposed hypotheses and a new matrix analysis. To test the proposed method, 3074 Taiwanese respondents, aged 15 years and older, were surveyed by telephone. I used the stratified simple random sampling method, with sample size allocation proportioned by the population distribution of 23 cities and counties (strata).

**Results:**

This study proposed the AWAG segment matrix to analyze the digital divide in eHealth services. First, awareness and want rates were divided into two levels at the middle point of 50%, and then the 2-dimensional cross of the awareness and want segment matrix was divided into four categories: opened group, desire-deficiency group, perception-deficiency group, and closed group. Second, according to the degrees of awareness and want, each category was further divided into four subcategories. I also defined four possible strategies, namely, hold, improve, evaluate, and leave, for different regions in the proposed matrix. An empirical test on two recently promoted eHealth services, the digital medical service (DMS) and the digital home care service (DHCS), was conducted. Results showed that for both eHealth services, the digital divides of awareness, want, and adoption existed across demographic variables, as well as between computer owners and nonowners, and between Internet users and nonusers. With respect to the analysis of the AWAG segment matrix for DMS, most of the segments, except for people with marriage status of Other or without computers, were positioned in the opened group. With respect to DHCS, segments were separately positioned in the opened, perception-deficiency, and closed groups.

**Conclusions:**

Adoption does not closely follow people’s awareness or want, and a huge digital divide in adoption exists in DHS and DHCS. Thus, a strategy to promote adoption should be used for most demographic segments.

## Introduction

Health care organizations are beginning to use the Internet in reaching a large part of the population in a cost-effective manner [1]. Several hundred thousand websites worldwide with varying qualities of health information are accessed and used by consumers and professionals [2]. The diffusion of broadband, wireless, and mobile Internet [3] has likewise influenced the traditional behavior of consumer activities, even in health care, thereby bringing about various social benefits. eHealth has changed the way health care is delivered and practiced [4]. For patients, who can also be viewed as consumers, eHealth presents an opportunity to change their relationship with providers, such as doctors and nurses [5]. The adoption of eHealth innovations can have a significant impact on the wellness of communities and populations [6].

eHealth services have improved access to health care in rural [7,8], suburban [9,10], and urban areas [11]. eHealth is particularly useful in linking specialists in academic health centers with health care professionals in areas short of facilities for patient care [12]. Following the rapid development of broadband Internet access services, the digital divide across demographic variables has become a huge social issue [13]. Affordable, high-speed wireless Internet access can be provided in rural and remote areas, bridging the gap between health care service and customers [14]. However, the availability of Internet access might cause another digital divide in eHealth between Internet users and nonusers, as well as between computer owners and nonowners. In fact, the digital divide in access to Internet technology has already caused inequalities in terms of health care [15].

In the last 10 years, researchers have begun discussing customer acceptance of eHealth services using the technology acceptance model [16,17] and the theory of planned behavior [16]. However, previous studies have simply discussed eHealth service adoption from the system design and improvement side, and scarcely explored the adoption of specific eHealth services. Studies examining the digital divide in eHealth services from a hierarchy-type viewpoint, such as which customers are adopting a new product or service, are rare. Therefore, the two main aims of this study are as follows: (1) from the customer segment viewpoint, to propose a new matrix analysis of the digital divide using the awareness, want, and adoption gap ratio (AWAG segment matrix), and thereafter compare the digital divide among different groups, and (2) to conduct an empirical study on specific eHealth services and show the practicability of the proposed matrix analysis.

## Methods

### Literature Review and Proposed Hypotheses

The digital divide relates not only to Internet access but also to the existence of a gap between people who can effectively use new information and communication tools, such as the Internet, and those who cannot [18]. The digital divide usually refers to access or usage; however, some studies have also identified two other divides: awareness [19] and demand (want) [20,21]. Barriers to the emergence of an equitable information society have led to the existence of the digital divide [22].

More differentiated use of the Internet across varying segments of a given population may result in the digital divide [22,23]. Moreover, demographic variables and socioeconomic status are factors influencing the digital divide [24,25]. Previous studies have indicated that the digital divide across demographic variables, including gender [20,26,27], age [20,26,28,29], education [26-31], income [26,27,29,32], marital status [26,30], geographic area [13,20,29,31,33], and ethnicity [20,31,34], are significant. Low-income [35] and elderly people, and those living in rural areas constitute the digitally underserved population [20,33], whereas people with higher education levels or of younger age are considered the digitally leading population [28,30]. Most studies have indicated that gender is no longer an influential factor in the digital divide [26,29,32,36]. However, some studies have asserted that, whereas males are most likely to access the Internet and play online games [26,37,38], females are most likely to use eHealth services [20]. Divorced people are more isolated than those who are married; this may contribute to a tendency not to use eHealth services [30].

The availability of a home computer is another factor used to predict an individual’s ability to access the Internet [24]. The ability to use a computer has been found to be associated with access to health-related information from the Internet [30]. People who are ill and have computer and Internet access desire specific information and may be more receptive to health information on managing their diseases [30,36,39].

Previous studies have shown that certain demographic variables and computer and Internet access are factors causing the digital divide, and that such a divide usually entails access or usage. Some studies have also identified two other divides: awareness [19] and demand (want) [20,21]. Thus, this research proposed three main hypotheses, with each having three subhypotheses, as follows:

H1: There exists an awareness divide in eHealth services across certain demographic variables, computer ownership, and Internet access.

H2: There exists a want divide in eHealth services across certain demographic variables, computer ownership, and Internet access.

H3: There exists an adoption divide in eHealth services across certain demographic variables, computer ownership, and Internet access.

The earliest and most well-known consumer purchasing decision process is attention–interest–desire–action, first proposed in the late 1800s and early 1900s [40,41]. Attention–interest–desire–action states that salespeople have to attract attention (cognition), maintain interest, and create desire (affect), leading to action (conation) [6,42]. Different models of consumer purchasing decisions consist of a sequence of mental stages or levels that consumers experience throughout the decision process [43-48]. Different studies have their own viewpoints, but most hierarchical models include six hierarchical stages: awareness, knowledge, liking, preference, conviction, and purchase. Some studies [47,49] have summarized the hierarchical stages of the consumer purchasing decision model into three stages: awareness, interest, and final decision. In the first stage, awareness, the consumer knows that an alternative exists but may not have the interest or sufficient information to understand its possible benefits. In the second stage, interest, the consumer is aware, develops some interest, and hence decides to learn more about the product. In this stage, the wants of consumers are singled out. In the last stage, final decision, the consumer takes an observable action, which is the purchase of a good or service or the sustained adoption of an innovation.

Some studies have mentioned that probabilities can be associated with the stages of the hierarchical models to show the ultimate behavioral impact of promotion [50-52]. Therefore, when evaluating the digital divide in eHealth services, the percentages or probabilities of awareness, want, and adoption, corresponding to the three main stages of purchasing decision, should be measured. Consumers’ want for eHealth services should also be created typically through promotion and education. However, the awareness of an eHealth service does not necessarily translate into choice or usage if there is a shortage of want. In other words, adoption does not occur if there is a shortage of awareness and want. The adoption rate of an e-service should be highly related to the corresponding awareness and want rates of individuals [53]. Thus, the following hypotheses were proposed:

H4: The adoption rate of a given eHealth service is bound to consumers’ corresponding awareness rate.

H5: The adoption rate of a given eHealth service is bound to consumers’ corresponding want rate.

From the end user’s viewpoint, Dixon [54] proposed the information technology adoption model (ITAM), which was compiled from several technology adoption models and incorporates end-user satisfaction. ITAM is based on a triangular structure of design–implementation–evaluation: it demonstrates the chicken-and-egg connection between the process of innovation design, and its implementation and evaluation. Referring to the concept of ITAM, the movement of a product or information between two subjects distinguishes technology push from consumer pull. Technology push, which is similar to the chicken analogy of ITAM, is mainly driven by research and development activities; and consumer pull, which is similar to the egg analogy of ITAM, is driven by external market forces. In the market, officers or suppliers *push* new products toward consumers. Meanwhile, consumers *pull* the goods or information they demand. A push marketing strategy is used when there is development or improvement on a product unknown to consumers. Given that there is no consumer demand in a product launch, the product and the information are pushed to consumers by distribution and promotion [55,56].

In a pull health care system, the patient requests the product and pulls it through the delivery channel [57]. Taking the online registration service of outpatients as an example, in the beginning, most patients did not request the service. The service was simply pushed to them through promotion by hospitals and the government. The patients were then made aware of such a service, and they considered whether they liked or needed it. Following an increased awareness of the online registration service for outpatients, designers have developed new functions needed by patients. This suggests that the demand from patients pulled the supply, as well as the corresponding improvements brought about by heightened awareness. Therefore, awareness and want gradually rise through the cycle of technology push and consumer pull. In general, want is initiated and raised when awareness spreads. The adoption rate is raised when the awareness of and want for a given e-service spread. In other words, the want rate should be lower than the awareness rate. However, according to the above discussion of pull and push, for some consumer segments, the want rate for new and innovative e-services is not necessarily always lower than their corresponding awareness rate. Thus, the following hypotheses were proposed:

H6: The want rate for a given eHealth service is not necessarily bound to consumers’ corresponding awareness rate.

H6-1: The want rate for a given eHealth service is bound to consumers’ corresponding awareness rate.

H6-2: The want rate for new and innovative e-services may be greater than consumers’ corresponding awareness rate.

Generally speaking, there is no adoption if there is no awareness. Higher awareness may bring higher want rates, but the intention of using some eHealth services will be low if there is a shortage of want. However, people having the potential need for an eHealth service will easily pay attention to the promotion and receive the information and, as such, may have a higher awareness rate than those who are not in need of the service. Thus, the following three hypotheses were proposed:

H7: Want rate, given awareness for each consumer segment, is higher than want rate with unawareness.

H8: Adoption rate, given want for each consumer segment, is higher than adoption rate without want.

H9: Awareness rate, given want for each consumer segment, is higher than awareness rate without want.

### AWAG Segment Matrix

According to H4 to H9, awareness and want have interactive influences on adoption rate. Therefore, when evaluating the digital divide in some e-services, the corresponding awareness and want rates should be considered separately. Based on the technology adoption lifecycle (bell curve), with a combination of innovators and early majority stages, the four types of adopters are segmented by three slightly adjusting adoption life cycle cumulative rates of 15%, 50%, and 85% [42]. The awareness and want rates should be high for innovators or early adopters—that is, following the rise of the innovation level, the adoption life cycle cumulative rates should move from low to high. The present study used the above three cumulative rates to segment and position groups.

**Figure 1:**
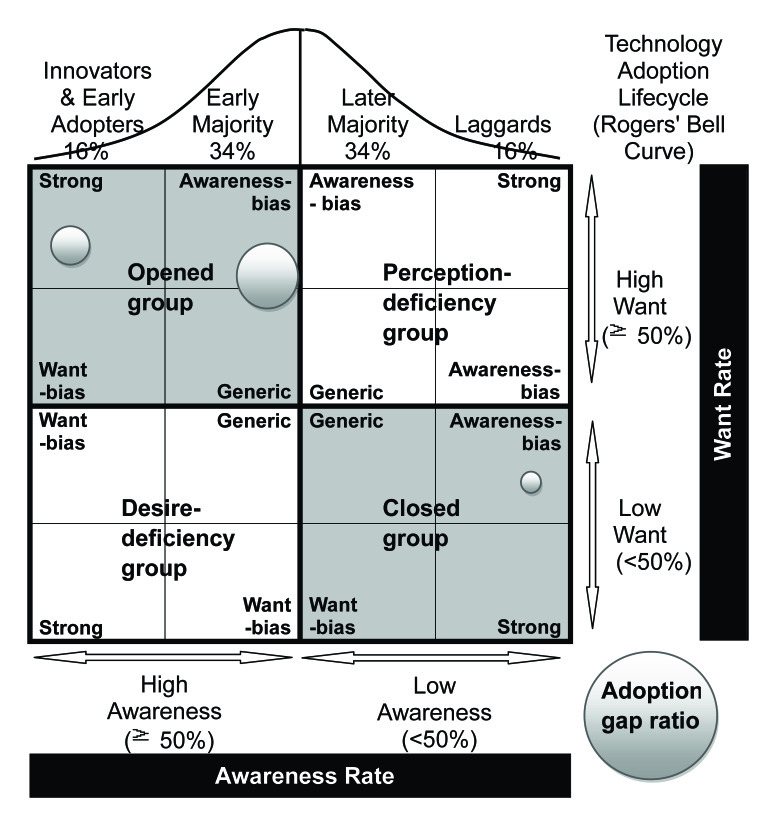
Awareness, want, and adoption gap ratio (AWAG) segment matrix.

First, awareness and want rates were divided into two levels at the middle point of 50%, and then the 2-dimensional cross of the AWAG segment matrix was divided into four categories: opened group, desire-deficiency group, perception-deficiency group, and closed group. Second, using the cumulative rate of 15% or 85%, each category was further divided into four subcategories. In the awareness–want segment matrix (Figure 1), the location of a group indicates its awareness and want rates for an eHealth service.

People categorized under the opened group are open to innovation. They are keen on seeking new information and are always on the lookout for something new and innovative. On the other hand, those who are categorized under the closed group are closed minded when it comes to innovation, and they lag behind in receiving new information. They are not interested in innovation and, as such, they usually resist trying something new. People categorized under the desire-deficiency group lack desire for innovation. Although they receive new information early, they are usually not interested in innovation and may resist trying something new. Meanwhile, those under the perception-deficiency group lack perception for innovation. Although they lag behind in receiving new information, they are still interested in innovation and always intend to try something new and innovative.

Each of the above four groups was further divided into four subgroups, according to the degrees of awareness and want, and based on a cumulative rate of 15% or 85%. For the opened group and closed group, the subgroups were strong, awareness-bias, want-bias, and generic. For the desire-deficiency group and perception-deficiency group, the subgroups were strong, generic, and want-bias or awareness-bias. The strong subgroup is the most open, closed, perception-deficient, or desire-deficient group. The generic subgroup is the least open, closed, perception-deficient, or desire-deficient group. There is some room to raise awareness for the awareness-bias subgroup and some room to raise want for the want-bias subgroup.

In the AWAG segment matrix, awareness and want are on the same level for four groups: strong opened group, generic opened group, generic closed group, and strong closed group. In these four groups, the awareness rate corresponds to the want rate. However, the opened degree for innovation decreases from left-up to right-down. For example, people under the strong opened group are innovators with the most open minds. Most of them already know about some innovations or new services, and they are full of want. On the opposite side, people under the strong closed group are laggards with the most closed minds. Most of them do not know or care about innovation or new services, and they are lacking in want.

Groups located on the left-down side of the downward-sloping 45° line have awareness rates greater than want rates. The groups located on the opposite side have inversed characteristics. The larger the distance beyond the 45° line, the greater the bias between awareness and want. For example, the group in the farthest left-down area is the strong desire-deficiency group. People belonging to the strong desire-deficiency group may not be the target of innovation. Although they have high awareness, they are short of want. Thus, any innovation promotion will not drive them to do something, and any promotion budget allocated to this group may be wasted. People belonging to the strong perception-deficiency group located at the farthest right-up, although high in want of innovation, are seriously ignored or may not have the capability to get information. Thus, they do not receive enough information on innovation. This group should be prioritized first, and more promotion efforts should be exerted on them.

The present study defined four possible strategies: hold, improve, evaluate, and leave [53,58,59]. The hold strategy maintains the good work for innovators, early adopters, and the early majority. The improve strategy includes three types of strategies: spread, create, and raise. The spread strategy promotes awareness by adjusting the communication channel or method for a segment. The create strategy identifies and forms new wants for a specific group. The raise strategy raises awareness or want for a segment. The evaluate strategy re-evaluates wants for a segment, and then further chooses from the leave or improve strategy. The leave strategy suggests not taking action in some specific groups because they are nontarget markets and should be left alone. Each group and the corresponding strategies and actions suggested are shown in Table 1.

**Table 1:** Prescriptions of the awareness, want, and adoption gap ratio (AWAG) segment matrix

Category	Subcategory	Strategy	Action	Current target market
Opened group	Strong	Hold	Keep up the good work.	Primary
	Awareness-bias	Hold and improve (raise)	Keep up the good work and keep raising the awareness.	Secondary
	Want-bias	Hold and improve (raise)	Keep up the good work and keep raising the want.	Tertiary
	Generic	Hold and improve (raise)	Keep up the good work and keep raising the awareness and want.	Tertiary
Closed group	Strong	Evaluate then leave or evaluate then improve (spread and create)	Evaluate the potential of the segment then choose an action between “maintain status quo” and “keep spreading the awareness or creating the want.”	Nontarget
	Awareness-bias	Evaluate then leave or evaluate then improve (spread)	Evaluate the potential of the segment then choose an action between “maintain status quo” and “keep spreading the awareness.”	Nontarget
	Want-bias	Evaluate then leave or evaluate then improve (create)	Evaluate the potential of the segment then choose an action between “maintain status quo” and “keep creating the want.”	Nontarget
	Generic	Evaluate then leave or evaluate then improve (raise)	Evaluate the potential of the segment then choose an action between “maintain status quo” and “keep raising the awareness or want.”	Nontarget
Desire-deficient group	Strong	Evaluate then leave or evaluate then improve (create)	Evaluate the potential of the segment then choose an action between “maintain status quo” and “keep creating the want.”	Nontarget
	Want-bias	Evaluate then leave or evaluate then improve (create)	Evaluate the potential of the segment then choose an action between “maintain status quo” and “keep creating the want.”	Nontarget
	Generic	Improve (create)	Keep creating the want.	Nontarget
Perception-deficient group	Strong	Improve (spread)	Keep spreading the awareness.	Potential target
	Awareness-bias	Improve (spread)	Keep spreading the awareness.	Potential target
	Generic	Improve (spread)	Keep spreading the awareness.	Potential target

**Figure 2:**
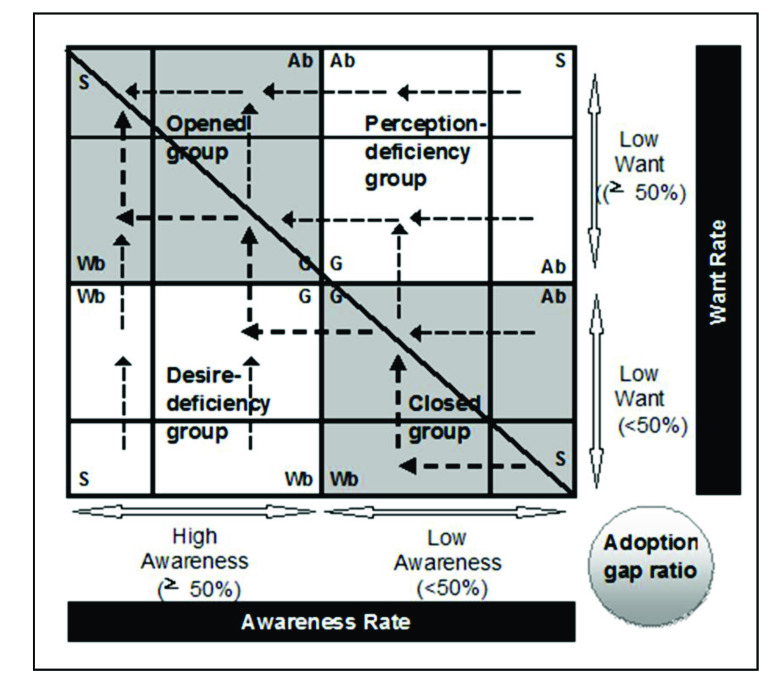
Improving directions for each region in the awareness, want, and adoption gap ratio (AWAG) segment matrix. Ab = awareness-bias; G = generic; S = strong; Wb = want-bias.

In using the AWAG segment matrix, managers should first re-evaluate the awareness and then the wants of some segments. If a segment is found to have low awareness, some promotion activities should be carried out, and follow-up should be conducted to raise the want. The e-services at the bottom right area should be pulled to the top left area, step by step, if possible or necessary. The suggested improvement direction for each group is shown in Figure 2. For example, the government of Taiwan has promoted the long-term management of physiological conditions since 2006, targeted at older people. At the beginning, news media were heavily used to raise awareness. Following an increased awareness, events demonstrating the benefit of long-term management of physiological conditions were held in some retirement communities, raising the want. When the want was identified, awareness spread more widely.

### Adoption Gap Ratio Analysis in the AWAG Segment Matrix

**Figure 3:**
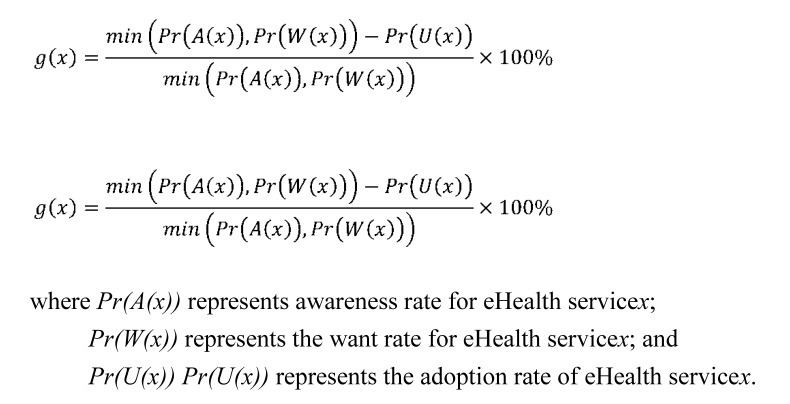
Equation for calculating the adoption gap ratio for service x.

Based on H4, H5, and H6, the adoption rate of a product or service is highly related to the corresponding awareness and want rates of consumers. However, even if consumers are aware of a new service or product, it does not follow that they will choose or use it. Therefore, the adoption rate is bound to the awareness and want rates. Under this situation, it is not proper to compare the adoption rates of products or services directly because they are under different levels of awareness and want (ie, the room for adoption promotion should be limited under current awareness and want). Therefore, Liang [53] proposed the adoption gap ratio analysis to explore the gap between adoption and awareness or want. The adoption gap ratio (*g(x)*gx) for service *x* is defined as shown in Figure 3.

The adoption gap ratio is the proportion of the adoption rate for a product or service thatcan be promoted under the current awareness or want rates. The range of the adoption gap ratio is from 0% to 100%. Among those who are already aware of or in want of an eHealth service, the adoption gate rate represents the percentage of people who have never used the service. The gate rate is 0% when the adoption rate is equal to the minimum value of the awareness and want rates. The gate rate is close to 100% when almost no one currently uses the product or service. Using the proposed adoption gap ratio, we can thus evaluate the effectiveness of adoption promotion more accurately. Several studies have found that perceived ease of use, perceived usefulness, and self-efficacy have direct effects on user attitude [60,61]. Therefore, when the gate rate is large, additional management and promotion strategies, such as enhancing the user friendliness of product or service functions or promoting adoption by education, should be used.

### Empirical Subjects

In 2002, 62.6% of hospitals in Taiwan had developed their own websites. Most of these hospitals agreed that applying Internet technology could improve service quality and work efficiency and that the Internet would have a huge influence on the delivery of medical websites [62]. In 2011, online reservation, electronic medical records, online inquiry for medical treatment, and online drug information services were already offered by all first-tier teaching hospitals and medical centers and by most second-tier teaching hospitals. The digital medical service (DMS) is now popular in Taiwan.

According to statistics compiled by the Ministry of the Interior in Taiwan, there were 1,490,801 elderly people in December 1996, representing 7.10% of Taiwan’s total population. This figure met the criteria of an old-age society set by the United Nations [63]. The elderly population has increased since then. In fact, it accounted for up to 10.21% of the population in 2010. As its population is aging fast, Taiwan has to cope with problems resulting from the continuing increase in the number of old people who need care. Therefore, the Department of Industrial Technology of the Ministry of Economic Affairs in Taiwan started planning and implementing a flagship project for technological innovation of health care service in 2006; the digital home care service (DHCS) is one of its major promotional services [64]. This project aims to bring essential care and benefits to elderly people, enabling them to live their lives with well-being, safety, convenience, and respect. Given that the DHCS has been promoted for only 5 years in Taiwan, the service has not yet gained in popularity.

To compare the digital divide on awareness, demand, and adoption of eHealth services in the different technological life cycles, the DMS and DHCS were selected as empirical subjects.

### Survey Method and Questionnaire

A telephone survey was conducted to evaluate the awareness of, want for, and use of DMS and DHCS. The survey method and questionnaire are presented in Multimedia Appendix 1.

## Results

### Sample Structure

In all, 3074 Taiwanese respondents aged 15 years and older were interviewed by telephone. The demographic, computer ownership, and Internet access profile of the respondents is shown in Table 2. The sample distributions of gender, age, and geographic area are as homogeneous as the population distribution (*P* > .05). Based on the survey, 90.66% of the respondents had computers at home and 70.07% had Internet access from anywhere.

**Table 2:** Profile of respondents: Demographic variables, computer ownership, and Internet access

Demographics			n	%
Total			3074	100.0
**Gender**				
	Male		1518	49.38
	Female		1556	50.62
**Age range (years)**				
	15−24		509	16.6
	25−34		621	20.2
	35−44		568	18.5
	45−54		565	18.4
	55−64		367	11.9
	≥65		444	14.4
**Education level**				
	Below primary school		384	12.5
	Junior high school		249	8.1
	Senior high school		1014	32.99
	Junior college		434	14.1
	University		847	27.6
	Graduate and above		146	4.8
**Marital status**				
	Single		1065	34.65
	Married or cohabiting		1876	61.03
	Other^a^		133	4.3
**Geographic area**				
	Northern Area		974	31.7
	Central Area		753	24.5
	Southern Area		663	21.6
	Eastern Area		87	3
	Taipei City		365	11.9
	Kaohsiung City		232	7.5
**Personal monthly income (US $)**				
		≤450	651	21.2
		451–650	211	6.9
		651–950	501	16.3
		951–1250	433	14.1
		1251–1550	279	9.1
		1551–2250	219	7.1
		≥2251	140	4.6
		Don’t know/no answer	641	20.8

**Family monthly income (US $)**				
		No income or unstable	104	3.4
		≤650	156	5.1
		651–1250	478	15.5
		1251–1850	561	18.3
		1851–2450	445	14.5
		2451–3050	316	10.3
		3051–4650	295	9.6
		≥4651	155	5.0
		Don’t know/no answer	563	18.3
**Computer ownership or Internet access**				
**Computer ownership**				
		Yes	2787	90.66
		No	286	9.3
**Internet access**				
		Yes	2154	70.07
		No	919	29.9

^a^ Divorced, separated, or widowed.

### Digital Divide Across the Demographic Variables

The awareness, want, and adoption rates of each demographic group for DMS and DHCS are shown in Table 3 and Table 4, respectively. There was a digital divide in awareness, want, and adoption in DMS across all the demographic variables, excluding gender (*P* < .05, see Multimedia Appendix 2: Table 5). Between females and males, there was no digital divide in awareness and want, whereas there was a digital divide in adoption. For DMS, the adoption rate among females is higher than that among males.

**Table 3:** Rates of awareness, want, and adoption by demographics and the corresponding regions in the awareness, want, and adoption gap ratio (AWAG) segment matrix for the digital medical service

			Stage							Region in awareness–want segment matrix^a^
			Awareness		Want		Adoption		Adoption gap ratio	
Variable	ID^b^	Item	n	%	n	%	n	%	%	
Total	T		2667	86.8	2390	77.8	1370	44.6	42.7	O_Wb
Gender	G1	Male	1323	87.1	1179	77.7	649	42.8	44.9	O_Wb
	G2	Female	1344	86.4	1211	77.9	721	46.3	40.5	O_Wb
Age range (years)	A1	15−24	402	78.9	399	78.4	189	37.0	52.8	O_G
	A2	25−34	579	93.2	524	84.4	343	55.2	34.5	O_Wb
	A3	35−44	539	94.9	470	82.8	282	49.6	40.1	O_Wb
	A4	45−54	510	90.3	454	80.3	245	43.3	46.0	O_Wb
	A5	55−64	321	87.5	280	76.4	143	39.0	49.0	O_Wb
	A6	≥65	315	71.1	263	59.3	169	38.0	35.9	O_G
Education level	E1	Below primary school	257	66.9	221	57.7	124	32.4	43.8	O_G
	E2	Junior high school	187	75.1	174	69.7	72	29.0	58.4	O_G
	E3	Senior high school	886	87.5	764	75.4	385	38.0	49.6	O_Wb
	E4	Junior college	410	94.5	372	85.9	223	51.4	40.1	O_S
	E5	University	784	92.6	720	84.9	469	55.3	34.9	O_Wb
	E6	Graduate and above	142	97.2	139	95.2	97	66.1	30.6	O_S
Marital status	M1	Single	918	86.2	863	81.1	466	43.8	46.0	O_Wb
	M2	Married or cohabiting	1685	89.9	1473	78.5	886	47.2	39.8	O_Wb
	M3	Other^c^	63	47.2	54	40.7	17	13.1	68.0	C_G
Geographic area	L1	Northern Area	868	89.2	775	79.6	442	45.4	43.0	O_Wb
	L2	Central Area	646	85.7	573	76.1	310	41.1	46.0	O_Wb
	L3	Southern Area	568	85.6	515	77.7	305	46.1	40.7	O_Wb
	L4	Eastern Area	76	86.9	74	84.6	37	41.9	50.5	O_Wb
	L5	Taipei City	341	93.5	304	83.4	194	53.3	36.1	O_Wb
	L6	Kaohsiung City	168	72.4	148	64.0	82	35.4	44.7	O_G
Personal monthly income (US $)	P1	≤450	520	79.9	474	72.9	265	40.7	44.2	O_G
	P2	451–650	181	85.7	161	76.3	90	42.9	43.7	O_Wb
	P3	651–950	460	91.9	414	82.7	240	47.8	42.2	O_Wb
	P4	951–1250	413	95.4	369	85.1	219	50.5	40.7	O_S
	P5	1251–1550	261	93.6	237	85.2	149	53.5	37.2	O_S
	P6	1551–2250	216	98.6	192	88.0	150	68.6	22.0	O_S
	P7	≥2251	137	97.8	124	88.5	62	44.4	49.9	O_S
	P8	Don’t know/no answer	479	74.8	419	65.3	195	30.4	53.5	O_G

Family monthly income (US $)	F1	No income or unstable	88	84.9	70	67.1	39	37.8	43.7	O_G
	F2	≤650	120	76.9	106	67.8	53	33.8	50.1	O_G
	F3	651–1250	398	83.2	347	72.6	178	37.3	48.6	O_G
	F4	1251–1850	501	89.2	462	82.3	257	45.8	44.3	O_Wb
	F5	1851–2450	412	92.6	379	85.1	239	53.6	37.0	O_S
	F6	2451–3050	296	93.5	274	86.9	176	55.6	36.0	O_S
	F7	3051–4650	280	94.8	253	85.9	164	55.8	35.1	O_S
	F8	≥4651	147	94.8	135	86.9	89	57.5	33.8	O_S
	F9	Don’t know/no answer	425	75.5	364	64.6	174	30.9	52.2	O_G
Computer ownership	C1	Yes	2496	89.6	2259	81.0	1305	46.8	42.2	O_Wb
	C2	No	170	59.5	132	46.0	65	22.7	50.8	D_G
Internet access	I1	Yes	1965	91.2	1816	84.3	1114	51.7	38.6	O_Wb
	I2	No	701	76.3	574	62.5	255	27.8	55.5	O_G

^a^ Groups are opened (O), desire-deficiency (D), perception-deficiency (P), and closed (C); regions are strong (S), generic (G), awareness-bias (Ab), and want-bias (Wb).

^b^ Item identifier.

^c^ Divorced, separated, or widowed.

**Table 4:** Rates of awareness, want, and adoption by demographics and the corresponding regions in the awareness, want, and adoption gap ratio (AWAG) segment matrix for the digital home care service

			Stage							Region in awareness–want segment matrix^a^
			Awareness		Want		Adoption		Adoption gap ratio	
Variable	ID^b^	Item	n	%	n	%	n	%	%	
Total	T		1563	50.9	2139	69.6	150	4.9	93.0	O_G
Gender	G1	Male	806	53.1	1055	69.5	84	5.5	92.1	O_G
	G2	Female	757	48.7	1084	69.7	66	4.3	93.9	P_G
Age range (years)	A1	15−24	250	49.1	366	71.9	31	6.1	91.5	P_G
	A2	25−34	318	51.2	440	70.8	32	5.1	92.8	O_G
	A3	35−44	315	55.5	415	73.1	23	4.0	94.5	O_G
	A4	45−54	334	59.2	432	76.5	23	4.1	94.6	O_G
	A5	55−64	173	47.2	246	67.1	15	4.1	93.9	P_G
	A6	≥65	173	39.0	239	53.9	27	6.0	88.9	P_G
Education level	E1	Below primary school	120	31.3	196	51.2	15	4.0	92.3	P_G
	E2	Junior high school	114	45.7	154	61.5	8	3.1	95.0	P_G
	E3	Senior high school	520	51.3	711	70.1	44	4.4	93.8	O_G
	E4	Junior college	262	60.4	330	76.1	20	4.5	94.0	O_G
	E5	University	451	53.3	636	75.1	55	6.4	91.4	O_G
	E6	Graduate and above	96	65.8	113	77.5	8	5.7	92.6	O_G
Marital status	M1	Single	541	50.8	765	71.8	62	5.8	92.0	O_G
	M2	Married or cohabiting	989	52.7	1325	70.6	86	4.6	93.5	O_G
	M3	Other^c^	34	25.1	49	37.0	2	1.7	95.3	C_G
Geographic area	L1	Northern Area	502	51.5	689	70.8	53	5.4	92.4	O_G
	L2	Central Area	410	54.4	536	71.2	44	5.8	91.8	O_G
	L3	Southern Area	323	48.8	443	66.7	29	4.4	93.5	P_G
	L4	Eastern Area	46	52.2	70	80.6	3	3.4	95.7	O_G
	L5	Taipei City	192	52.6	270	74.1	10	2.6	96.4	O_G
	L6	Kaohsiung City	91	39.4	131	56.5	12	5.1	91.0	P_G
Personal monthly income (US $)	P1	<450	277	42.6	422	64.8	33	5.1	92.1	P_G
	P2	451–650	117	55.6	146	69.0	13	6.2	91.0	O_G
	P3	651–950	258	51.5	358	71.4	24	4.8	93.3	O_G
	P4	951–1250	228	52.7	332	76.7	23	5.4	93.0	O_G
	P5	1251–1550	174	62.6	206	74.0	15	5.3	92.9	O_G
	P6	1551–2250	135	61.5	178	81.2	14	6.2	92.4	O_G
	P7	≥2251	93	66.6	112	79.6	12	8.7	89.0	O_G
	P8	Don’t know/no answer	280	43.7	387	60.4	16	2.5	95.8	P_G

Family monthly income (US $)	F1	No income or unstable	40	38.3	60	57.7	1	0.6	99.0	P_G
	F2	≤650	71	45.3	92	58.7	5	3.2	94.6	P_G
	F3	651–1250	210	44.0	330	69.2	24	5.0	92.8	P_G
	F4	1251–1850	320	57.0	416	74.1	26	4.7	93.7	O_G
	F5	1851–2450	246	55.3	325	73.0	29	6.4	91.2	O_G
	F6	2451–3050	171	54.2	237	74.9	17	5.5	92.7	O_G
	F7	3051–4650	182	61.8	232	78.7	22	7.6	90.4	O_G
	F8	≥4,651	87	56.2	113	72.8	12	8.0	89.1	O_G
	F9	Don’t know/no answer	236	41.9	334	59.3	14	2.4	95.9	P_G
Computer ownership	C1	Yes	1473	52.9	2026	72.7	143	5.1	92.9	O_G
	C2	No	90	31.4	114	39.7	6	2.3	94.3	C_G
Internet access	I1	Yes	1192	55.3	1615	75.0	125	5.8	92.3	O_G
	I2	No	371	40.4	524	57.0	25	2.7	95.3	P_G

^a^ Groups are opened (O), desire-deficiency (D), perception-deficiency (P), and closed (C); regions are strong (S), generic (G), awareness-bias (Ab), and want-bias (Wb).

^b^ Item identifier.

^c^ Divorced, separated, or widowed.

With respect to the digital divide in DHCS, except for gender, the *P* values of the chi-square independent tests for awareness and want are all less than .05 (see Multimedia Appendix 2: Table 6), indicating that there was a digital divide in awareness and want in DHCS across all the demographic variables, excluding gender. Between females and males, there was no digital divide in want, whereas there was a digital divide in awareness. The awareness rate of males was higher than that of females. The above results support hypotheses H1, H2, and H3.

### Adoption Rate is Always Bound to Awareness and Want Rates

In the paired proportion test between adoption and awareness or want rates for the demographic groups, there are significant differences between adoption and awareness or want rates across the demographic groups (*P* < .05, see Multimedia Appendix 2: Table 7 and Table 8). Given that all the adoption rates are less than the awareness and want rates (Table 3, Table 4), the results support hypotheses H4 and H5.

### Want Rate is Not Necessarily Bound to Awareness Rate

In the paired proportion test between awareness and want rates for the demographic groups, there are significant differences between awareness and want rates for most demographic groups (*P* < .05, see Multimedia Appendix 2: Table 7 and Table 8). However, the awareness rates are significantly greater than the want rates for DMS, which is an existing eHealth service in Taiwan. The want rates are significantly greater than the awareness rates for DHCS, which is a new eHealth service in Taiwan (Table 3, Table 4). This indicates that the want rate is not necessarily dependent on the awareness rate. Thus, hypotheses H6, H6-1, and H6-2 are supported by the above results.

### Conditional Relationship among Awareness, Want, and Adoption Rates

In the independent proportion test of want rates between unawareness and awareness for DMS and DHCS, there are significant differences for most demographic groups (*P* < .05, see Multimedia Appendix 2: Table 9 and Table 10). Furthermore, for all the demographic groups, want rates given unawareness are less than want rates given awareness. Thus, H7 is supported. For most of the demographic groups, the adoption or awareness rates under “wanted” are greater than those under “unwanted” for DMS and DHCS. Thus, hypotheses H8 and H9 are mostly supported.

### AWAG Segment Matrix Analysis

**Figure 4:**
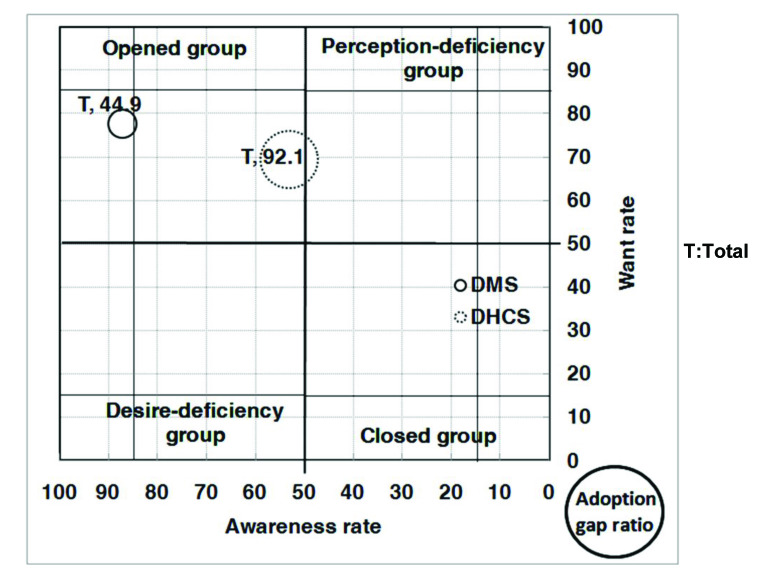
Awareness, want, and adoption gap ratio (AWAG) segment matrix for the digital medical service (DMS) and digital home care service (DHCS).

**Figure 5:**
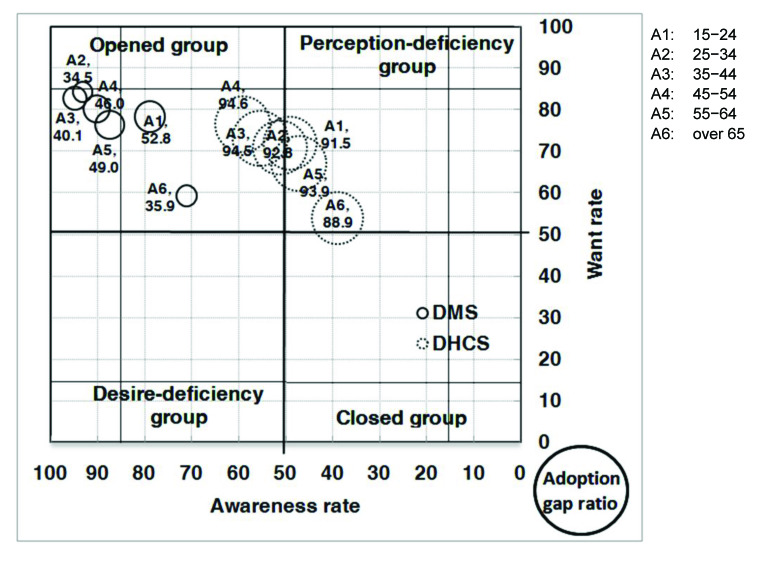
Awareness, want, and adoption gap ratio (AWAG) segment matrix by age (A; years) for the digital medical service (DMS) and digital home care service (DHCS).

**Figure 6:**
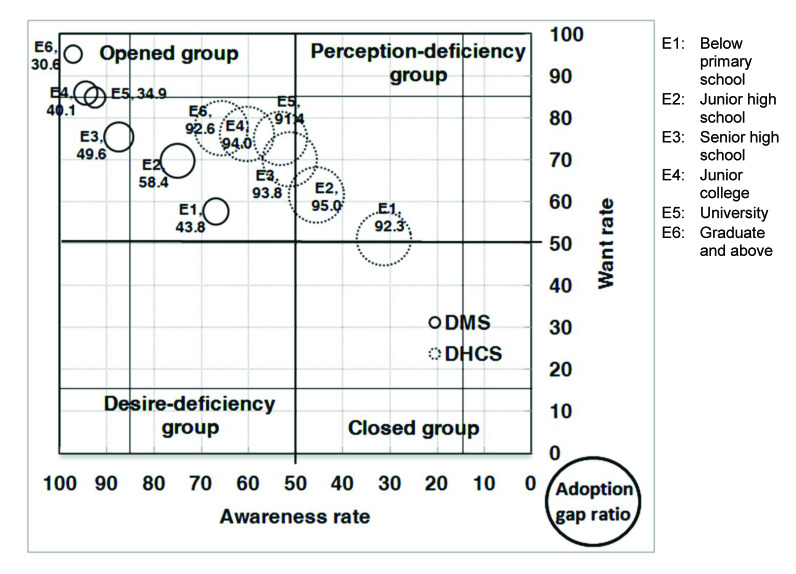
Awareness, want, and adoption gap ratio (AWAG) segment matrix by educational level (E) for the digital medical service (DMS) and digital home care service (DHCS).

**Figure 7:**
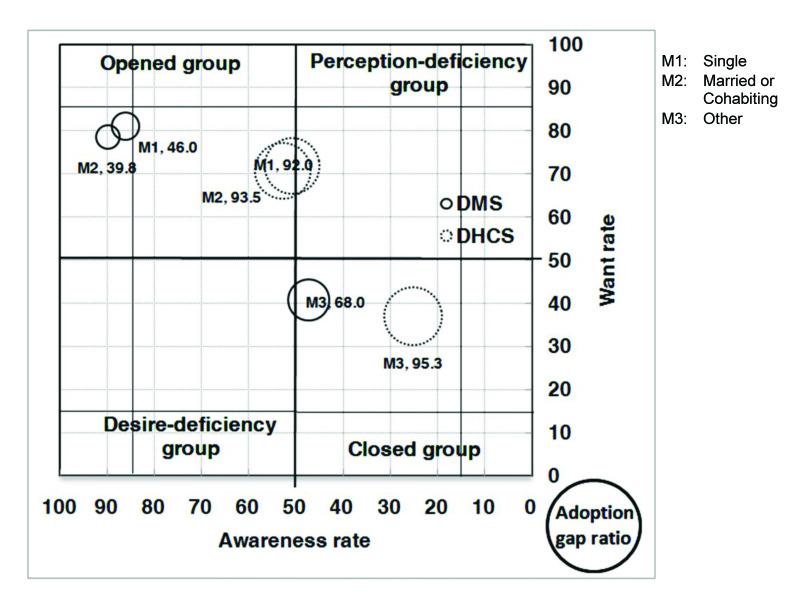
Awareness, want, and adoption gap ratio (AWAG) segment matrix by marital status (M) for the digital medical service (DMS) and digital home care service (DHCS).

**Figure 8:**
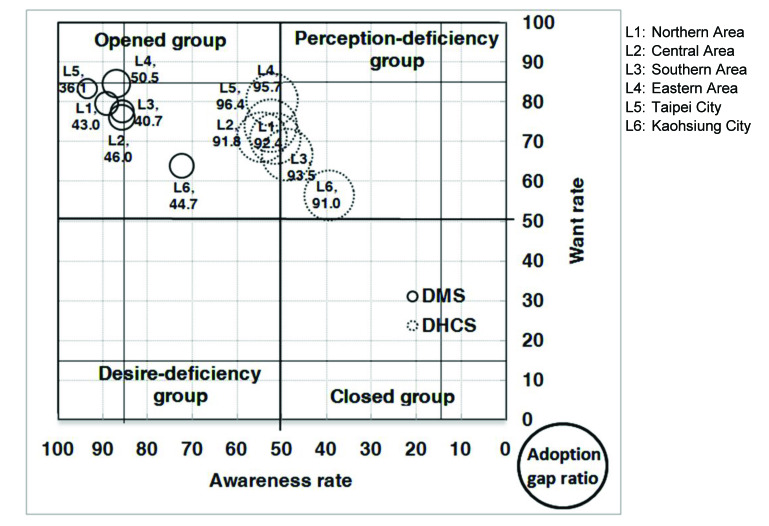
Awareness, want, and adoption gap ratio (AWAG) segment matrix by geographic area (L) for the digital medical service (DMS) and digital home care service (DHCS).

**Figure 9:**
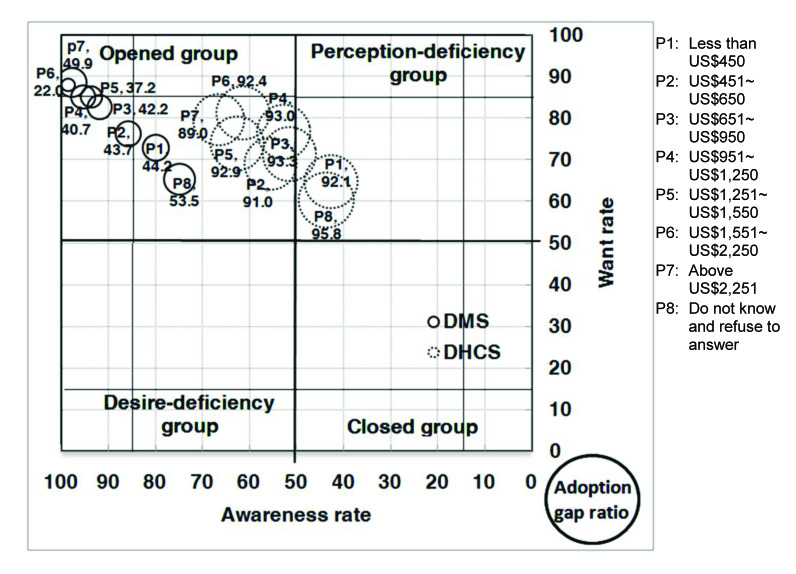
Awareness, want, and adoption gap ratio (AWAG) segment matrix by personal monthly income (P) for the digital medical service (DMS) and digital home care service (DHCS).

**Figure 10:**
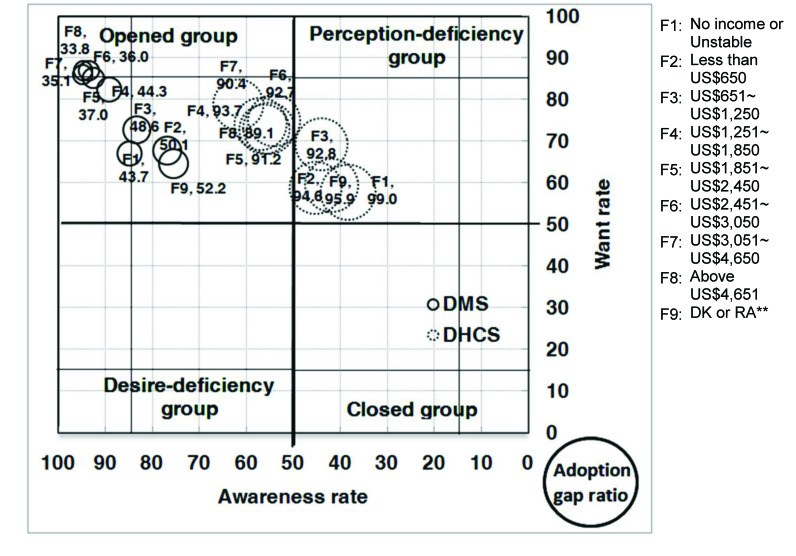
Awareness, want, and adoption gap ratio (AWAG) segment matrix by family monthly income (F) for the digital medical service (DMS) and digital home care service (DHCS). DK = don't know; RA = refused to answer.

**Figure 11:**
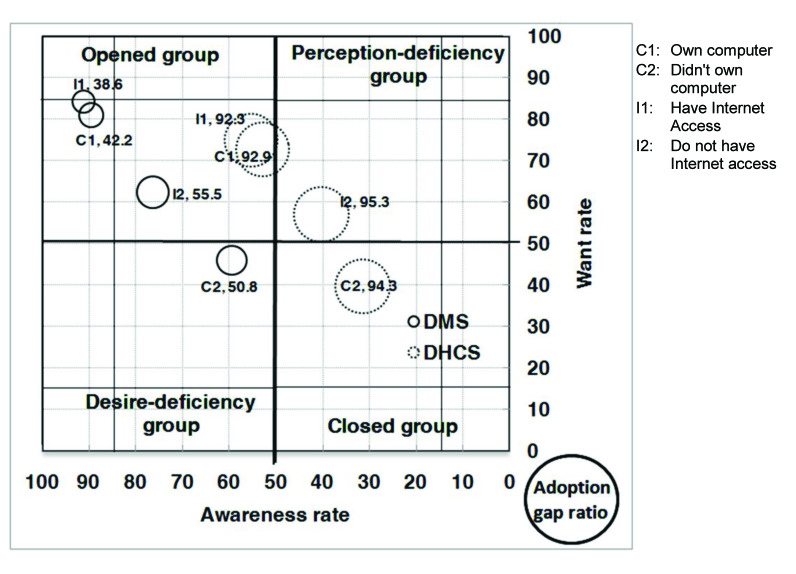
Awareness, want, and adoption gap ratio (AWAG) segment matrix by computer ownership (C) and Internet access (I) for the digital medical service (DMS) and digital home care service (DHCS).

With respect to the analysis of the AWAG segment matrix for DMS, most of the segments, except for people without a computer or with marriage status of divorced, separated, or widowed, are positioned under the opened group. This is not surprising given that DMS was already well established in Taiwan. However, although most of the segments belong to the opened group, their degrees of openness are different. In general, individuals with high levels of personal and family monthly incomes, as well as those with education levels of graduate and above, belong to the strong opened group, whereas those who are either younger or older, have low education and family monthly income levels, are living in Kaohsiung City, or have no computer or Internet access belong to the generic opened group. Those without a computer belong to the generic desire-deficiency group with relatively low want, indicating that they may have high awareness of DMS, but their needs may not be identified. People with a marriage status of divorced, separated, or widowed belong to the generic closed group. With relatively low DMS awareness, they may be encountering some barriers in obtaining information (Figure 4, Figure 5, Figure 6, Figure 7, Figure 8, Figure 9, Figure 10, Figure 11, Table 3).

The adoption gap ratios of segments for DMS range from 22.0% to 68.0%. All segments still have room to promote adoption. Among people with a marriage status of Other, 68% of those who were aware or in want of DMS did not adopt the service. The adoption gap ratios are near or above 50% among people aged between 25 and 34 or between 55 and 64 years; having education levels of junior high school or senior high school; having marriage status of Other; living in the eastern area; having personal monthly incomes above US $2251 and family monthly incomes less than 1250, or did not know or refused to answer questions on personal or family monthly income; and had no computer or Internet access. These findings indicate a huge potential area where DMS adoption can be promoted.

With respect to analysis of the AWAG segment matrix for DHCS, segments are separately positioned in the opened, perception-deficiency, and closed groups. Segments of females; aged between 15 and 24, 55 and 64, and above 65 years; having education levels below primary school or junior school; living in the southern area or Kaohsiung City; having personal monthly incomes of less than US $450, or did not know or refused to answer questions on personal income; having minimal family monthly incomes that were unstable or less than US $1250, or did not know or refused to answer questions on family income; or had no Internet access are positioned in the generic perception-deficiency group. This indicates that the awareness of DHCS for those segments is relatively low. People with marital status of divorced, separated, or widowed, as well as those without a computer, belong to the generic closed group. Both their awareness and want rates are relatively low. Other segments not mentioned above all belong to the generic opened group (Figure 4, Figure 5, Figure 6, Figure 7, Figure 8, Figure 9, Figure 10, Figure 11, Table 3). The gap ratios of all segments are all greater than or near 90%. Given that DHCS is a new eHealth service in Taiwan, there are huge potential areas where DHCS adoption can be promoted for all segments.

## Discussion

The results of this study show that digital divides in DMS and DHCS exist across certain demographic variables. In addition, the study has proven that the want rate is not always bound to the awareness rate. The want rate is usually bound to the awareness rate for existing services, such as DMS. However, DHCS is an innovative e-service in Taiwan; thus, for this service, the want rate is higher than the awareness rate. This study has also proven that awareness and want have reciprocal effects. Adoption may be pulled with rising awareness and want. A higher awareness rate may result in a higher want rate, and people in need of some eHealth services have a higher awareness rate than those who are not in need of the service. Therefore, the innovation diffusion process should start from awareness, followed by want, and awareness and want will gradually rise through the cycle of technology push and consumer pull, and pull adoption.

Using AWAG segment matrix analysis led to several conclusions. With respect to DMS, most segments belong to the opened group. Based on the adoption gap ratio analysis, all the gap values are higher than 22%, signifying that there is room for raising strategies for DMS adoption.

For DMS, segments with high levels of personal and family monthly incomes, as well as segments with education levels of graduate and above, all belong to the strong opened group and are the primary target markets. The marketing strategy of “hold” and the action of “keep up the good work” are suggested. Compared with other segments, the adoption gap ratio for those with personal monthly incomes above US $2251 is the highest and is near 50%. This segment should be ranked first in terms of adoption promotion strategies.

Segments with members who are either younger or older, have low education levels, have low family monthly income levels, live in Kaohsiung City, and have no Internet access belong to the generic opened group for DMS. For these, the “hold and improve strategy” and the action of “keep up the good work and keep raising the awareness and want” are recommended. These segments constitute the third target market for DMS. Other segments, except for people without a computer and with marriage status of Other, constitute the secondary target market for DMS. Thus, the “hold and improve strategy” with the action of “keep up the good work and keep raising the awareness” is suggested.

People without computers belong to the generic desire-deficiency group and are nontarget markets for DMS. The marketing strategy of “improve (create)” and the action of “keep creating the want” are suggested. The adoption gap ratio for this segment is 50.8%; in this segment, half of those who do not adopt DMS are in want of DMS. Thus, the adoption promotion strategy should also be used at once. People with marriage status of Other belong to the generic closed group. The adoption gap ratio for this group is the highest; in this segment, 68% of those who do not adopt DMS are in want of DMS. Thus, the “evaluate then improve (raise)” strategy and the actions of “evaluate the potential of the segment then keep raising the awareness or want” and “promote the adoption” are suggested.

With respect to DHCS, half of the segments belong to the opened group, and one-third of the segments belong to the perception-deficiency group. According to the adoption gap ratio analysis, because DHCS is a new eHealth service in Taiwan, all the gap values are higher than or near 90%, indicating that there is a huge room for raising DHCS adoption.

Segments with members who are female, young, late-middle aged, or elderly; have low education levels; live in the southern area or Kaohsiung City; have low or unstable personal or family incomes or refuse to answer questions on income; or without Internet access belong to the generic perception-deficiency group for DHCS. These segments are potential target markets for DHCS, and the marketing strategy “improve (spread)” and the action of “keep spreading the awareness” are suggested.

People who are divorced, separated, or widowed, or without computers belong to the generic closed group for DHCS. These two segments are nontarget markets for DHCS, and the “evaluate then leave or evaluate then improve (raise)” strategy and the action of “evaluate the potential of the segment then choose an action between “maintain status quo” and “keep raising the awareness or want” should be used. Other segments for DHCS all belong to the generic opened group. These constitute the third target market for DHCS. The “hold and improve strategy” and the action of “keep up the good work” and “keep raising the awareness and want” are thus suggested.

### Conclusion

This study has proposed the AWAG segment matrix analysis and analyzed the digital divides in DMS and DHCS across different demographic groups. From the results of this study, the digital divide in awareness and want across different demographic groups can be easily observed by cross-segmenting the awareness and want rates. Marketing strategies have also been clearly established. The adoption gap ratio between adoption and awareness or want rates is large for DMS and even larger for DHCS. These indicate that adoption does not closely follow peoples’ awareness or want, and that a huge digital divide in adoption exists in DHS and DHCS. Adoption education and promotion programs should therefore be used.

For marketing managers in business, government, or other related institutions, the AWAG segment matrix provides a simple and clear method for analyzing the digital divides and differentiating between target and nontarget markets. Moreover, it helps managers adjust their market strategies and allocate their budget more effectively from an objective and customer-oriented viewpoint.

### Suggestions for Future Study

For further research, the AWAG segment matrix can be revised by adding “satisfaction with eHealth service” into the analysis. The AWAG segment matrix can also be extended to analyze differential concerns on information security among the different segments mentioned in this study.

## Multimedia Appendix 1

Survey methodology and questionnaire.

## Multimedia Appendix 2

Tables 5-11.

## Abbreviations

AWAG: awareness, want, and adoption gap ratio
DHCS: digital home care service
DMS: digital medical service
ITAM: information technology adoption model
