# Supplementary material for: Matrix Analysis of the Digital Divide in eHealth Services Using Awareness, Want, and Adoption Gap
Source: J Med Internet Res. 2012 Feb 13;14(1):e11. doi: 10.2196/jmir.1670 (PMC3374548; doi:10.2196/jmir.1670)
Supplement: Supplementary file 1 [file jmir_v14i1e11_app1.pdf]

Multimedia Appendix 1: the survey methodology and questionnaire

1. Survey Methodology

Survey Method	Computer Assisted Telephone Interviewing Survey
Sample Number	3,074 valid samples
Target	Residents in Taiwan aged 15 and above
Population	20,373,096 residents in 20 cities/counties in Taiwan.
Sampling Method	A stratified simple random sampling was used by separating the population elements into strata composed of the 23 cities/counties in Taiwan. The sample size of each stratum was determined by using the proportional allocation method.

2.Questionnaire

The Usage Survey on Digital Medical Services

Good day, this is the Survey and Statistics Analysis Research and Consult Center of Fu Jen Catholic University. We are making a telephone survey on the usage regarding digital medical services. I would like to ask you some questions. Please join me for just a few minutes. Thank you!

- **Are you a citizen of R.O.C.?**
  □1. Y □2. N (End the interview.)

- **Are you 15 years old or older?**
  □1. Y □2. N (End the interview.)

- **Does this telephone number belong to a household or household-business mixed?**
  □1. Y □2. N (End the interview.)

- **Your telephone was automatically selected by a computer. Can you please tell me the county or city this telephone number belongs to?**
  □1. Northern Area □2. Central Area □3. Southern Area
  □4. Eastern Area □5. Taipei City □6. Kaohiung City
  □7. Refused to answer (End the interview.)

1. **Have you ever heard of services or contents regarding 'Digital medical service (DMS)'? (For example: on-line reservation, electronic medical records, on-line inquiry for medical treatment, on-line drug information, and so on)**
   □1. Y □2. N (Do not need to interview question 3)

2. **Are you in want of 'Digital Medical Service (DMS)'?**
   □1. Y □2. N

3. **Have you ever used 'Digital Medical Service (DMS)'?**
   □1. Y □2. N

4. **Have you ever heard of services or contents regarding 'Digital home care service (DHCS)'? (For example: long-term management of physiological conditions, voice drug reminder, abnormal records, and active care and contact)**
   □1. Y □2. N (Do not need to interview question 6)

5. **Are you in want of 'Digital home care service (DHCS)'?**
   □1. Y □2. N

6. Have you ever used 'Digital home care service (DHCS)'?

□1. Y □2. N

7. Do you have a computer at home?

□1. Y □2. N
□3. Do not know or refused to answer

8. Have you ever used the Internet?

□1. Y □2. N (Do not need to interview question 9)
□3. Do not know or refused to answer (Do not need to interview question 9)

9. What method do you use to connect to the Internet? (Multiple choices)

□1. Broadband (includes dial-up) □2. Wireless □3. Mobile

10. What is your highest educational attainment?

□1. Primary and below □2. Junior middle school
□3. Senior and professional high school □4. Junior college
□5. Undergraduate college □6. Postgraduate and above

11. What is your age? (Check the appropriate box.)

□1. 15–24 y □2. 25–34 y □3. 35–44 y
□4. 45–54 y □5. 55–64 y □6. 65 y and above

12. What is your current marital status?

□1. Single □2. Married or cohabitating
□3. Other (includes divorced or separated, widow or widower)
□4. Do not know or refused to answer

13. How many children do you have?

□1. Non □2. Have children (How many?_______)
□3. Do not know or refused to answer

14. What is your personal monthly income?

□1. Less than US$450 □2. US$451~ US$650
□3. US$651~ US$950 □4. US$951~ US$1,250
□5. US$1,251~ US$1,550 □6. US$1,551~ US$2,250
□7. Above US$2,251 □8. Do not know or refused to answer

15. What is your average monthly family income?

□1. No income or Unstable □2. Less than US$650
□3. US$651~ US$1,250 □4. US$1,251~ US$1,850
□5. US$1,851~ US$2,450 □6. US$2,451~ US$3,050
□7. US$3,051~ US$4,650 □8. Above US$4,651
□9. Do not know or refused to answer

16. What is the gender of the interviewee?

□ 1. Male □ 2. Female
