# Supplementary material for: Matrix Analysis of the Digital Divide in eHealth Services Using Awareness, Want, and Adoption Gap
Source: J Med Internet Res. 2012 Feb 13;14(1):e11. doi: 10.2196/jmir.1670 (PMC3374548; doi:10.2196/jmir.1670)
Supplement: Supplementary file 2 [file jmir_v14i1e11_app2.pdf]

Multimedia Appendix 2

Table 5. Results of chi-squared independent tests for the digital divide in DMS

**Demographics**		Digital Medical Service (DMS)		
		**Awareness H1**	**Want H2**	**Adoption H3**
Gender	Chi-squared	.41	.02	3.99
	df	1	1	1
	P-value	**.524**	**.889**	.046*
Age Group	Chi-squared	182.88	113.05	58.51
	df	5	5	5
	P-value	<.001*	<.001*	<.001*
Educational Level	Chi-squared	222.63	169.38	140.66
	df	5	5	5
	P-value	<.001*	<.001*	<.001*
Marital Status	Chi-squared	195.65	113.76	60.03
	df	2	2	2
	P-value	<.001*	<.001*	<.001*
Living Area	Chi-squared	62.20	37.39	124.60
	df	5	5	7
	P-value	<.001*	<.001*	<.001*
Personal monthly Income	Chi-squared	198.37	119.17	117.89
	df	7	7	8
	P-value	<.001*	<.001*	<.001*
Family Monthly Income	Chi-squared	135.86	133.92	23.78
	df	8	8	5
	P-value	<.001*	<.001*	<.001*
Computer owned	Chi-squared	204.77	184.64	60.88
	df	1	1	1
	P-value	<.001*	<.001*	<.001*
Internet Access	Chi-squared	125.25	177.90	149.83
	df	1	1	1
	P-value	<.001*	<.001*	<.001*

Data resource: This research

P-value means the p value of the Fisher’s exact test or of Pearson Chi-squared test.

H1, H2, H3 are alternative hypotheses. H1: There exists an awareness divide in e-health services across certain demographic variables, computer ownership, and internet access. H2: There exists a want divide in e-health services across certain demographic variables, computer ownership, and internet access. H3: There exists an adoption divide in e-health services across certain demographic variables, computer ownership, and internet access.

Table 6. Results of chi-squared independent tests for the digital divide in DHCS

Demographics		Digital Home Care Service (DHCS)		
		**Awareness H1**	**Want H2**	**Adoption H3**
Gender	Chi-squared	5.99	.01	2.74
	df	1	1	1
	P-value	.014*	**.920**	**.098**
Age Group	Chi-squared	48.03	69.92	5.19
	df	5	5	5
	P-value	<.001*	<.001*	**.394**
Educational Level	Chi-squared	91.67	93.92	7.84
	df	5	5	5
	P-value	<.001*	<.001*	**.165**
Marital Status	Chi-squared	37.50	70.88	5.65
	df	2	2	2
	P-value	<.001*	<.001*	**.059**
Living Area	Chi-squared	17.94	31.55	6.51
	df	5	5	5
	P-value	.003*	<.001*	**.259**
Personal monthly Income	Chi-squared	72.11	67.13	14.17
	df	7	7	7
	P-value	<.001*	<.001*	.048*
Family Monthly Income	Chi-squared	64.08	68.00	21.08
	df	8	8	8
	P-value	<.001*	<.001*	.007*
Computer owned	Chi-squared	47.46	133.49	5.17
	df	1	1	1
	P-value	<.001*	<.001*	.023*
Internet Access	Chi-squared	57.75	98.20	13.19
	df	1	1	1
	P-value	<.001*	<.001*	<.001*

Data resource: This research

P-value means the p value of the Fisher’s exact test or of Pearson Chi-squared test.

H1, H2, H3 are alternative hypotheses. H1: There exists an awareness divide in e-health services across certain demographic variables, computer ownership, and internet access. H2: There exists a want divide in e-health services across certain demographic variables, computer ownership, and internet access. H3: There exists an adoption divide in e-health services across certain demographic variables, computer ownership, and internet access.

Table 7. P-values of the paired proportion tests for H4, H5, and H6 in DMS

Demographics			Digital Medical Service (DMS)		
			H4	H5	H6
Variables	ID	Items	P-value	P-value	P-value
Total	T		<.001	<.001	<.001
Gender	G1	Male	<.001	<.001	<.001
	G2	Female	<.001	<.001	<.001
Age Level	A1	15−24	<.001	<.001	<.001
	A2	25−34	<.001	<.001	<.001
	A3	35−44	<.001	<.001	<.001
	A4	45−54	<.001	<.001	<.001
	A5	55−64	<.001	<.001	<.001
	A6	over 65	<.001	<.001	<.001
Educational Level	E1	Below primary school	<.001	<.001	**.105**
	E2	Junior high school	<.001	<.001	<.001
	E3	Senior high school	<.001	<.001	<.001
	E4	Junior college	<.001	<.001	<.001
	E5	University	<.001	<.001	<.001
	E6	Graduate and above	<.001	<.001	**.339**
Marital Status	M1	Single	<.001	<.001	<.001
	M2	Married or Cohabiting	<.001	<.001	<.001
	M3	Other*	<.001	<.001	**.100**
Living Area	L1	Northern Area	<.001	<.001	<.001
	L2	Central Area	<.001	<.001	<.001
	L3	Southern Area	<.001	<.001	<.001
	L4	Eastern Area	<.001	<.001	**.657**
	L5	Taipei City	<.001	<.001	<.001
	L6	Kaohsiung City	<.001	<.001	.003
Personal Monthly Income	P1	Less than US$450	<.001	<.001	.001
	P2	US$451~ US$650	<.001	<.001	.004
	P3	US$651~ US$950	<.001	<.001	<.001
	P4	US$951~ US$1,250	<.001	<.001	<.001
	P5	US$1,251~ US$1,550	<.001	<.001	.001
	P6	US$1,551~ US$2,250	<.001	<.001	<.001
	P7	Above US$2,251	<.001	<.001	.002
	P8	DK or RA**	<.001	<.001	<.001
Family Monthly Income	F1	No income or Unstable	<.001	<.001	.001
	F2	Less than US$650	<.001	<.001	.030
	F3	US$651~ US$1,250	<.001	<.001	<.001
	F4	US$1,251~ US$1,850	<.001	<.001	<.001
	F5	US$1,851~ US$2,450	<.001	<.001	<.001
	F6	US$2,451~ US$3,050	<.001	<.001	.003
	F7	US$3,051~ US$4,650	<.001	<.001	<.001
	F8	Above US$4,651	<.001	<.001	.005
	F9	DK or RA**	<.001	<.001	<.001
Computer Ownership	C1	Own	<.001	<.001	<.001
	C2	Didn't own	<.001	<.001	<.001
Internet Access	I1	Yes	<.001	<.001	<.001
	I2	No	<.001	<.001	<.001

Data resource: This research

* Including divorced, separated, widower, or widow

**'DK or RA' represents "do not know" or "refuse to answer."

H4, H5, H6 are alternative hypotheses. H4: The adoption rate of a given e-health service is bound to consumers' corresponding awareness rate. H5: The adoption rate of a given e-health service is be bound to consumers' corresponding want rate. H6: The want rate for a given e-health service is not necessarily bound to consumers' corresponding awareness rate.

Table 8. P-values of the paired proportion tests for H4, H5, and H6 in DHCS

Demographics			Digital Home Care Service (DHCS)		
			H4	H5	H6
Variables	ID	Items	P-value	P-value	P-value
Total	T		<.001	<.001	<.001
Gender	G1	Male	<.001	<.001	<.001
	G2	Female	<.001	<.001	<.001
Age Level	A1	15−24	<.001	<.001	<.001
	A2	25−34	<.001	<.001	<.001
	A3	35−44	<.001	<.001	<.001
	A4	45−54	<.001	<.001	<.001
	A5	55−64	<.001	<.001	<.001
	A6	over 65	<.001	<.001	<.001
Educational Level	E1	Below primary school	<.001	<.001	<.001
	E2	Junior high school	<.001	<.001	<.001
	E3	Senior high school	<.001	<.001	<.001
	E4	Junior college	<.001	<.001	<.001
	E5	University	<.001	<.001	<.001
	E6	Graduate and above	<.001	<.001	.028
Marital Status	M1	Single	<.001	<.001	<.001
	M2	Married or Cohabiting	<.001	<.001	<.001
	M3	Other*	<.001	<.001	.005
Living Area	L1	Northern Area	<.001	<.001	<.001
	L2	Central Area	<.001	<.001	<.001
	L3	Southern Area	<.001	<.001	<.001
	L4	Eastern Area	<.001	<.001	<.001
	L5	Taipei City	<.001	<.001	<.001
	L6	Kaohsiung City	<.001	<.001	<.001
Personal Monthly Income	P1	Less than US$450	<.001	<.001	<.001
	P2	US$451~ US$650	<.001	<.001	.003
	P3	US$651~ US$950	<.001	<.001	<.001
	P4	US$951~ US$1,250	<.001	<.001	<.001
	P5	US$1,251~ US$1,550	<.001	<.001	.003
	P6	US$1,551~ US$2,250	<.001	<.001	<.001
	P7	Above US$2,251	<.001	<.001	.015
	P8	DK or RA**	<.001	<.001	<.001
Family Monthly Income	F1	No income or Unstable	<.001	<.001	.001
	F2	Less than US$650	<.001	<.001	.004
	F3	US$651~ US$1,250	<.001	<.001	<.001
	F4	US$1,251~ US$1,850	<.001	<.001	<.001
	F5	US$1,851~ US$2,450	<.001	<.001	<.001
	F6	US$2,451~ US$3,050	<.001	<.001	<.001
	F7	US$3,051~ US$4,650	<.001	<.001	<.001
	F8	Above US$4,651	<.001	<.001	.001
	F9	DK or RA**	<.001	<.001	<.001
Computer Ownership	C1	Own	<.001	<.001	<.001
	C2	Didn't own	<.001	<.001	.009
Internet Access	I1	Yes	<.001	<.001	<.001
	I2	No	<.001	<.001	<.001

Data resource: This research

* Including divorced, separated, widower, or widow

**'DK or RA' represents "do not know" or "refuse to answer."

H4, H5, H6 are alternative hypotheses. H4: The adoption rate of a given e-health service is bound to consumers' corresponding awareness rate. H5: The adoption rate of a given e-health service is be bound to consumers' corresponding want rate. H6: The want rate for a given e-health service is not necessarily bound to consumers' corresponding awareness rate.

Table 9. P-values of the two independent proportion tests for H7, H8, and H9 in DMS

Demographics/ Computer ownership/ Internet access		Digital Medical Service								
		H7			H8			H9		
		Want rate (%)			Adoption rate (%)			Awareness rate (%)		
		Given unaware	Given Aware	P-value	Given Unwanted	Given wanted	P-value	Given Unwanted	Given wanted	P-value
Total		46.65	82.52	<.001	9.83	54.49	<.001	68.22	92.05	<.001
Gender	Male	51.30	81.55	<.001	10.99	51.90	<.001	71.95	91.50	<.001
	Female	42.37	83.47	<.001	8.68	57.01	<.001	64.53	92.59	<.001
Age Level	15−24	60.87	83.09	<.001	8.61	44.87	<.001	61.80	83.64	<.001
	25−34	61.01	86.05	.002	16.92	62.33	<.001	83.17	95.12	.003
	35−44	78.48	83.03	**.572**	6.07	58.66	<.001	93.65	95.19	**.529**
	45−54	65.96	81.80	.021	15.99	50.04	<.001	83.22	92.00	.021
	55−64	42.83	81.19	<.001	10.65	47.72	<.001	69.77	93.01	<.001
	over 65	16.02	76.95	<.001	4.58	60.92	<.001	40.28	92.19	<.001
Educational Level	Below primary school	22.09	75.30	<.001	4.12	53.16	<.001	39.04	87.32	<.001
	Junior high school	44.28	78.11	<.001	3.51	40.11	<.001	54.17	84.15	<.001
	Senior high school	57.46	77.96	<.001	9.22	47.35	<.001	78.31	90.44	<.001
	Junior college	69.91	86.78	**.096**	16.56	57.15	<.001	88.28	95.51	**.095**
	University	65.98	86.46	.001	18.22	61.89	<.001	83.26	94.24	.002
	Graduate and above	77.43	95.76	**.500**	20.10	68.40	.008	86.87	97.75	**.424**
Marital Status	Single	62.24	84.10	<.001	12.22	51.16	<.001	72.48	89.42	<.001
	Married or Cohabiting	46.86	82.09	<.001	9.54	57.55	<.001	74.93	93.95	<.001
	Other*	13.60	71.01	<.001	5.18	24.50	.004	23.11	82.38	<.001
Living Area	Northern Area	49.65	83.25	<.001	10.73	54.22	<.001	73.26	93.25	<.001
	Central Area	57.73	79.20	<.001	9.89	50.90	<.001	74.73	89.18	<.001
	Southern Area	46.65	82.94	<.001	9.44	56.55	.002	65.62	91.39	<.001
	Eastern Area	76.29	85.90	**.407**	10.41	47.58	.002	79.78	88.19	**.407**
	Taipei City	46.03	85.98	.001	15.12	60.93	<.001	78.93	96.42	.002
	Kaohsiung City	18.06	81.50	<.001	4.29	52.89	<.001	37.17	92.20	<.001
Personal monthly Income	Less than US$450	50.81	78.43	<.001	8.19	52.75	<.001	63.53	85.98	<.001
	US$451~ US$650	47.32	81.10	.001	4.77	54.78	<.001	68.27	91.13	.006
	US$651~ US$950	64.49	84.32	.015	4.73	56.84	<.001	83.35	93.68	.016
	US$951~ US$1,250	82.52	85.26	**.736**	14.08	56.89	<.001	94.55	95.51	**.736**
	US$1,251~ US$1,550	75.12	85.87	**.332**	23.70	58.71	<.001	89.30	94.38	**.324**
	US$1,551~ US$2,250	56.12	88.42	**.432**	33.80	73.38	<.001	94.72	99.08	**.341**
	Above US$2,251	76.07	88.82	**.487**	27.39	46.59	**.135**	95.34	98.08	**.487**
	DK or RA**	30.35	77.11	<.001	6.33	43.18	<.001	49.37	88.29	<.001
Family Monthly Income	No income or Unstable	50.55	70.00	**.134**	5.62	53.54	<.001	77.39	88.65	**.176**
	Less than US$650	40.94	75.84	<.001	1.88	48.96	<.001	57.72	86.07	.001
	US$651~ US$1,250	43.42	78.50	<.001	6.65	48.84	<.001	65.37	89.98	<.001
	US$1,251~ US$1,850	69.36	83.92	.022	8.52	53.84	<.001	81.28	90.92	.022
	US$1,851~ US$2,450	61.70	86.95	.007	11.60	61.00	<.001	80.97	94.62	.008
	US$2,451~ US$3,050	70.45	88.01	**.111**	16.59	61.49	<.001	85.47	94.77	**.110**
	US$3,051~ US$4,650	77.18	86.41	**.313**	26.60	60.53	<.001	91.58	95.34	**.414**
	Above US$4,651	43.38	89.29	.042	33.81	61.10	.020	77.41	97.39	**.051**
	DK or RA**	29.32	76.09	<.001	7.35	43.78	<.001	51.00	88.87	<.001
Computer Ownership	Own	58.59	83.64	<.001	12.46	54.85	<.001	77.21	92.45	<.001
	Didn't own	16.72	66.01	<.001	0.82	48.27	<.001	37.44	85.28	<.001
Internet Access	Yes	62.73	86.38	<.001	14.88	58.58	<.001	79.15	93.47	<.001
	No	32.71	71.71	<.001	4.87	41.56	<.001	57.50	87.58	<.001

Data resource: This research

* Including divorced, separated, widower, or widow

**'DK or RA' represents "do not know" or "refuse to answer."

H7, H8, H9 are alternative hypotheses. H7: Want rate, given awareness for each consumer segment, is higher than want rate with unawareness. H8: Adoption rate, given want for each consumer segment, is higher than adoption rate without want. H9: Awareness rate, given want for each consumer segment, is higher than awareness rate without want.

Table 10. P-values of the two independent proportion tests for H7, H8, and H9 in DHCS

Demographics/ Computer ownership/Internet access		Digital Home Care Service								
		H7			H8			H9		
		Want rate			Adoption rate			Awareness rate		
		Given unaware	Given aware	P-value	Given Unwanted	Given wanted	P-value	Given Unwanted	Given wanted	P-value
Total		64.15	74.86	<.001	1.21	6.48	<.001	42.06	54.71	<.001
Gender	Male	62.34	75.86	<.001	1.11	7.43	<.001	42.07	57.95	.001
	Female	65.77	73.81	.001	1.32	5.55	<.001	42.06	51.56	<.001
Age Level	15−24	69.67	74.31	**.244**	1.85	7.73	.001	44.93	50.69	**.244**
	25−34	65.96	75.50	.009	2.03	6.35	.006	42.99	54.53	.009
	35−44	69.73	75.87	**.104**	0.55	5.26	<.001	49.84	57.56	**.104**
	45−54	76.72	76.35	**.919**	2.10	4.71	**.108**	59.55	59.05	**.919**
	55−64	61.39	73.47	**.103**	1.15	5.52	.013	38.09	51.73	.013
	over 65	42.89	71.18	<.001	0.00	11.08	<.001	24.40	51.49	<.001
Educational Level	Below primary school	41.05	73.29	<.001	0.00	7.74	<.001	17.13	44.88	<.001
	Junior high school	53.54	71.06	.004	0.58	4.64	.032	34.40	52.78	.004
	Senior high school	67.66	72.41	**.100**	0.97	5.83	<.001	47.34	52.99	.099
	Junior college	75.56	76.41	**.841**	1.77	5.41	.044	59.56	60.67	**.840**
	University	72.04	77.70	**.059**	2.84	7.65	.002	47.61	55.14	**.058**
	Graduate and above	78.34	77.12	**.868**	0.00	7.40	.003	67.00	65.43	**.868**
Marital Status	Single	67.56	75.99	.002	1.94	7.28	<.001	43.29	53.71	.002
	Married or Cohabiting	66.66	74.22	<.001	1.00	6.08	<.001	46.33	55.42	<.001
	Other*	24.01	75.68	<.001	0.00	4.68	**.130**	9.70	51.41	<.001
Living Area	Northern Area	69.33	72.15	**.334**	1.32	7.09	<.001	49.12	52.53	**.334**
	Central Area	66.09	75.42	.005	2.23	7.31	.001	46.36	57.65	.005
	Southern Area	59.30	74.56	<.001	0.99	6.03	<.001	37.31	54.49	<.001
	Eastern Area	80.76	80.41	**.967**	3.31	3.46	**.976**	52.67	52.11	<.001
	Taipei City	67.65	79.93	.008	0.00	3.56	.002	40.76	56.72	**.957**
	Kaohsiung City	44.51	74.90	<.001	0.00	8.98	<.001	22.73	52.25	.007
Personal monthly Income	Less than US$450	57.97	74.10	<.001	0.65	7.53	<.001	31.41	48.72	<.001
	US$451~ US$650	65.84	71.53	**.377**	3.36	7.48	**.190**	51.03	57.59	**.377**
	US$651~ US$950	65.33	77.17	.003	0.39	6.50	<.001	41.19	55.69	.003
	US$951~ US$1,250	74.81	78.37	**.384**	2.93	6.10	**.140**	48.93	53.88	**.384**
	US$1,251~ US$1,550	70.30	76.17	**.291**	2.21	6.34	.091	57.33	64.47	**.282**
	US$1,551~ US$2,250	84.91	78.91	**.257**	3.40	6.84	**.412**	69.02	59.71	**.260**
	Above US$2,251	80.48	79.16	**.865**	0.00	10.98	<.001	68.00	66.19	**.856**
	DK or RA**	54.52	67.85	.001	0.45	3.86	.001	35.46	49.17	.001
Family Monthly Income	No income or Unstable	48.34	72.91	.011	0.00	1.02	**.507**	24.56	48.35	.012
	Less than US$650	44.10	76.44	<.001	0.98	4.74	**.143**	25.85	58.91	<.001
	US$651~ US$1,250	65.97	73.29	**.083**	1.40	6.61	.002	38.15	46.62	.083
	US$1,251~ US$1,850	66.86	79.63	**1<.001**	0.00	6.29	<.001	44.90	61.23	.001
	US$1,851~ US$2,450	71.91	73.88	**.642**	2.18	7.98	.004	53.48	55.95	**.642**
	US$2,451~ US$3,050	73.50	76.05	**.603**	4.14	5.94	**.545**	51.63	55.00	**.603**
	US$3,051~ US$4,650	80.81	77.39	**.487**	2.56	8.91	.022	65.56	60.74	**.487**
	Above US$4,651	65.53	78.53	**.077**	0.00	10.94	<.001	44.41	60.58	**.072**
	DK or RA**	54.11	66.52	0.003	0.50	3.74	.005	34.50	47.01	.003
Computer Ownership	Own	69.71	75.31	0.001	1.39	6.56	<.001	47.76	54.78	.001
	Didn't own	26.93	67.58	<.001	0.45	5.03	.033	16.91	53.50	<.001
Internet Access	Yes	72.86	76.67	0.044	2.10	7.04	<.001	51.59	56.59	.044
	No	48.86	69.08	<.001	0.00	4.74	<.001	29.06	48.93	<.001

Data resource: This research

* Including divorced, separated, widower, or widow

**'DK or RA' represents "do not know" or "refuse to answer."

H7, H8, H9 are alternative hypotheses. H7: Want rate, given awareness for each consumer segment, is higher than want rate with unawareness. H8: Adoption rate, given want for each consumer segment, is higher than adoption rate without want. H9: Awareness rate, given want for each consumer segment, is higher than awareness rate without want.
